# The Role of CC10 Combined With CysLTs in Predicting Subsequent Wheezing in RSV‐Infected Bronchiolitis

**DOI:** 10.1155/carj/6698429

**Published:** 2026-07-14

**Authors:** Meng Deng, Qiujin Yuan, Sisi Chen, Zhengzhen Tang

**Affiliations:** ^1^ Department of Pediatrics, The Third Affiliated Hospital of Zunyi Medical University (The First People’s Hospital of Zunyi), Zunyi, Guizhou, China; ^2^ Chongqing Engineering Research Center of Pharmaceutical Sciences, Chongqing Medical and Pharmaceutical College, Chongqing, China, cqmu.edu.cn

**Keywords:** CC10, CysLTs, RSV, subsequent wheezing

## Abstract

**Objective:**

To clarify the predictive role of club cell 10‐kDa protein (CC10) and cysteinyl leukotrienes (CysLTs) for subsequent wheezing after RSV infection.

**Methods:**

From October 1, 2022, to October 31, 2023, children diagnosed with RSV‐induced bronchiolitis hospitalized in the pediatric ward were included. Nasopharyngeal aspirates (NPAs) were obtained on the day of admission. NPAs from hospitalized children without respiratory infections served as the control group. We analyzed the correlation between CC10 and CysLTs levels in NPAs and disease severity. Follow‐up assessments were conducted to observe the subsequent wheezing.

**Results:**

There were 169 hospitalized infants diagnosed with RSV‐bronchiolitis, and 22 healthy controls matched for age were selected. The levels of CC10 in the NPAs were significantly lower in the RSV group (219.4 ± 83.1 pg/mL) than those in the control group (284.1 ± 68.1 pg/mL; *p* < 0.001). Levels of CysLTs were significantly greater in the RSV group (346.3 ± 98.1 pg/mL) than in the control group (288.3 ± 82.5 pg/mL; *p* < 0.05). Pearson correlation analysis showed that CC10 protein level was negatively correlated with severity (*r* ^2^ = 0.34, *p* < 0.001). Conversely, the level of CysLTs was positively correlated with severity (*r* ^2^ = 0.09, *p* < 0.001). 142 cases were completed with follow‐up within a year after discharge. 47 cases (33.1%) had experienced subsequent wheezing, and 95 cases (66.9%) did not have subsequent wheezing. The multivariate logistic regression analysis indicates a higher likelihood of subsequent wheezing in children with CysLTs levels over 335.3 pg/mL (OR = 5.496), allergy history (OR = 3.466), and longer ventilation duration (OR = 1.364).

**Conclusions:**

While CC10 and CysLTs levels correlated with the severity of RSV‐bronchiolitis, only CysLTs, along with allergy history and duration of ventilation, showed a suggestive link to subsequent wheezing, but further validation is needed.

## 1. Introduction

Respiratory syncytial virus (RSV) is the most common viral pathogen causing acute lower respiratory tract infections in infants globally. Annually, approximately 33.8 million children under 5 years old suffer from acute lower respiratory infections caused by RSV infection [1]. Moreover, up to 30% of children with severe RSV infections develop recurrent wheezing later in life [2]. To date, no biological markers have been identified that can predict the subsequent wheezing following RSV infection.

Respiratory epithelial cells constitute the first line of defense against viral infections and are closely associated with disease severity and long‐term prognosis [3]. Our previous studies have demonstrated that the number of club cells, which are a subgroup of lung epithelial cells, is significantly reduced in BALB/c mice infected with RSV [4]. At the same time, the level of club cell 10‐kDa protein (CC10) secreted by club cells into the bronchoalveolar lavage fluid (BALF) was significantly reduced after RSV infection [5].

CC10 is a 10‐kDa, globular, nonglycosylated secretory globulin with a high baseline secretion level [6]. It exerts anti‐inflammatory effects by inhibiting the activity of Cytosolic phospholipase A2 (cPLA2) and plays a protective role in airway injury diseases [7]. cPLA2 phosphorylates and degrades membrane phospholipids to produce arachidonic acid (AA), which is further metabolized through the lipoxygenase (LOX) pathway to generate the unstable intermediate Leukotriene A4 (LTA4). LTA4 is subsequently converted into two distinct leukotriene products—cysteinyl leukotrienes (CysLTs) and Leukotriene B4 (LTB4) [8]. In our previous studies, we found that the level of LOX in lung tissue was elevated, along with an increase in downstream CysLTs, while LTB4 levels remained unchanged on Day 5 after RSV infection in BALB/c mice. After administration of recombinant CC10, the levels of CysLTs were reduced, accompanied by improvements in airway inflammation and airway hyperresponsiveness (AHR) [9]. This suggests that CC10 can inhibit the role of downstream CysLTs in the leukotriene pathway in the airway inflammatory response following RSV infection.

Therefore, this study aims to further investigate the levels of CC10 and downstream CysLTs in the leukotriene pathway in nasopharyngeal aspirates (NPAs) from children with RSV‐induced bronchiolitis. We will observe whether these levels are correlated with the clinical features of the children and the subsequent wheezing in the later stages. The study will also determine the value of these two factors in predicting subsequent wheezing following RSV infection.

## 2. Materials and Methods

### 2.1. Ethics Statement

The acquisition and use of the NPAs were approved by the Institutional Review Boards of the Third Affiliated Hospital of Zunyi Medical University (the First People’s Hospital of Zunyi) (Permit number: 2022‐1‐45, Guizhou, China). Written informed consent was obtained from the guardians of the patients. All procedures were carried out in accordance with the approved guidelines and in compliance with the principles of the Declaration of Helsinki.

### 2.2. Infant NPAs and Clinical Data

From October 1, 2022, to October 31, 2023, 169 hospitalized infants diagnosed with RSV‐bronchiolitis (the first episode of acute expiratory wheezing in a child < 2 years of age) in the Department of Pediatrics, the Third Affiliated Hospital of Zunyi Medical University (the First People’s Hospital of Zunyi), Guizhou, China, were enrolled in the RSV group. Infants with Down syndrome, a history of wheezing, or cardiac or pulmonary pathology were excluded. Within the first days of admission, NPAs were prospectively collected from all subjects. RSV was detected using a direct single fluorescent assay (D3 DFA Respiratory Viruses Screening and ID Kit; Diagnostic Hybrids, Hannover, Germany). When a virus other than RSV was detected in the infants, they were not included. Those considered positive for bacterial infection by the published criteria [10] were excluded as well. According to the clinical severity scoring method described by Wang et al. [11], patients whose severity score was < 5 points were regarded as mild, children with a score of 5–8.9 points were considered moderate, while severity scores ≥ 9 were considered to be in the severe group. The data collected included the demographics (age, sex, delivery, preterm, feeding history, and allergy history), clinical characteristics (fever and length of stay), and investigations (blood routine test, liver, and renal function tests). CC10 is almost exclusively pulmonary; trace amounts of the protein are found in the fallopian tube and prostate [7]. Therefore, control diseases with potential involvement of these sites were excluded. There were 22 ICU control children, comprising 9 with convulsions and 13 who had undergone elective surgery without respiratory infection and were under postoperative ICU observation. None of these diseases involved the genital system, so CC10 levels were unaffected. NPA samples were obtained only because airway clearance was clinically required after convulsions or anesthesia. During collection, any specimen that failed to reach 0.5 mL was discarded. The levels of CC10 and CysLTs in NPAs were compared in both the RSV group and the control group. The relationships between these two factors and disease severity were analyzed, and the association between these factors and subsequent wheezing was followed up.

### 2.3. NPAs Preparation

0.5 mL of NPA specimen from every infant was collected on the day of admission and then transferred to another tube, and 2 mL of 0.1% dithiothreitol was added. The samples were fully mixed, vortexed for 15 s, and rocked on a bench rocker for 15 min, followed by centrifugation at 2500 r.p.m. for 10 min at 4°C. The supernatants were collected and stored at −80°C for cytokine estimation.

### 2.4. ELISA Analysis

The concentrations of CC10 and CysLTs in the supernatants of the infants’ NPAs were determined using CC10 and CysLTs ELISA kits according to the manufacturer’s instructions (Jingmei, Jiangsu, China).

### 2.5. Follow‐Up

Telephone follow‐up was conducted to assess the subsequent wheezing within a year after discharge in children with RSV‐induced bronchiolitis. An episode was recorded when parents could audibly detect wheezing or when a physician, during a respiratory illness visit, reported wheezing or auscultated wheeze. The correlation between CC10 and CysLTs levels and the subsequent wheezing was analyzed.

### 2.6. Statistical Analysis

All analyses were performed using SPSS 29.0. Statistical significance was assessed by the *t*‐test and one‐way ANOVA for the levels of CC10 and CysLTs and expressed as the means ± SE. Qualitative data were analyzed using the *χ* ^2^ test and were described by frequency and percent. The correlations were detected by the Pearson correlation. The receiver operating characteristic curve (ROC) was used to evaluate the efficacy of CC10 and CysLTs as biomarkers for subsequent wheezing after RSV infection, and the area under the curve (AUC) and the optimal threshold were calculated, respectively. The multivariate analyses were performed using stepwise logistic regression and stepwise linear regression to evaluate the independent predictors of subsequent wheezing in infants with RSV‐bronchiolitis. The results are presented as odds ratios (ORs), regression coefficients (*β*), and 95% confidence intervals (95% CIs).

## 3. Results

### 3.1. Patient Characteristics

From October 1, 2022, to October 31, 2023, 169 hospitalized infants diagnosed with RSV‐bronchiolitis were included in the RSV group. 22 infants were included in the control group. The median age of the RSV‐infected infants was 5.7 months (interquartile range [IQR]: 2.4–13.0 months), and 115 (68.0%) were male. The median age in the control group was 7.0 months (IQR: 4.0–10.5 months), and 14 (63.6%) were male. There were no significant differences in age and sex distribution between the RSV group and the control group (*p* > 0.05).

### 3.2. Levels of CC10 and CysLTs in NPAs in the RSV Group and the Control Group

**FIGURE 1:**
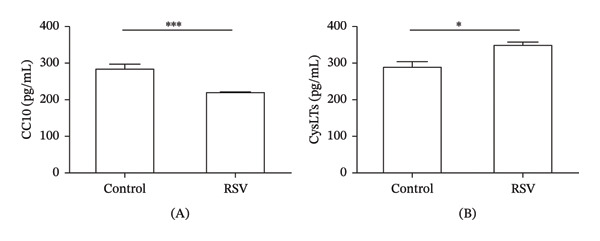
The levels of club cell 10‐kDa protein (CC10) and cysteinyl leukotrienes (CysLTs) in the nasopharyngeal aspirates (NPAs) of infants with respiratory syncytial virus (RSV) bronchiolitis. The concentrations of CC10 and CysLTs in the NPAs were assessed using ELISA. (A) CC10 levels in NPAs. ^∗∗∗^ *p* < 0.001. (B) CysLTs levels in NPAs. ^∗^ *p* < 0.05, hospitalized patients with RSV‐associated bronchiolitis (*n* = 169) compared to infants without respiratory infections (*n* = 22).

The levels of CC10 in the NPAs were significantly lower in hospitalized infants with RSV‐bronchiolitis (219.4 ± 83.1 pg/mL) than in infants without lower respiratory tract infections (284.1 ± 68.1 pg/mL; *p* < 0.001; Figure 1A). The levels of CysLTs were significantly greater in the RSV group (346.3 ± 98.1 pg/mL) than those in the control group (288.3 ± 82.5 pg/mL; *p* < 0.05; Figure 1B).

### 3.3. Clinical Characteristics of Children Diagnosed With RSV‐Bronchiolitis With Varying Severity

The 169 patients were divided into 3 groups according to the clinical severity score described by Wang et al. [11], resulting in the following groups: 61 (36.1%) cases of mild disease, 56 (33.1%) cases of moderate disease, and 52 (30.8%) cases of severe disease. Clinical characteristics are shown in Table 1. The age of children in the moderate (6.5 months [2.0–6.5 months]) and severe groups (5.5 months [2.5–5.5 months]) was younger than that in the mild group (9.3 months [5.5–17.0 months]). The rate of preterm birth in the severe group (19.2%) was higher than that in the moderate group (8.9%). The proportion of previous allergic disease in the severe group (15.4%) was higher than that in the moderate group (8.9%) and the mild group (1.6%). The hospital stay of the mild group was 6.3 ± 2.1 days, the moderate group was 8.9 ± 3.3 days, and the severe group was 12.2 ± 5.0 days. The hospital stay of the severe group was longer than that of the mild group and the moderate group. The above differences were statistically significant (*p* < 0.05). The laboratory examination of the RSV group and the control group were compared. The ratio of neutrophils (*N*%) in the severe group (43.7% ± 20.0%) was higher than that in the mild group (35.1% ± 16.4%) and the moderate group (32.7% ± 17.8%, *p* = 0.006). The lymphocyte ratio (*L*%) of the severe group (44.6% ± 16.6%) was lower than that of the mild group (53.1% ± 15.7%) and the moderate group (55.8% ± 17.4%, *p* = 0.002). The hemoglobin of the severe group (108.4 ± 10.9 g/L) was lower than that of the mild group (116.9 ± 10.9 g/L) and the moderate group (118.1 ± 27.7 g/L, *p* = 0.017).

**TABLE 1:** Clinical characteristics of children diagnosed with RSV‐bronchiolitis with varying severity.

	Mild group (*n* = 61)	Moderate group (*n* = 56)	Severe group (*n* = 52)	χ^2^/*H*/*F*/*t*	*p*
Male/female	42/19	40/16	33/19	0.82	0.665
Age (months), median (IQR)	9.3 (5.5, 17.0)	6.5 (2.0, 6.5)^a^	5.5 (2.5, 5.5)^a^ ^,^ ^b^	11.33	0.003
Premature birth (< 37 weeks) (%)	6 (9.8)	5 (8.9)	10 (19.2)	3.22	0.200
Vaginal delivery (%)	42 (68.9)	27 (48.2)^a^	42 (80.8)^b^	13.10	0.001
Breastfeeding (%)	4 (6.5)	7 (12.5)^a^	15 (28.8)^a^	46.91	< 0.001
History of allergy (%)	1 (1.6)	5 (8.9)	8 (15.4)^a^	7.03	0.030
Fever (%)	22 (36.1)	16 (28.6)	17 (32.7)	0.75	0.668
Length of stay (days)	6.3 ± 2.1	8.9 ± 3.3^a^	12.2 ± 5.0^a^ ^,^ ^b^	6.01	0.003
White blood cells (× 10^9^/L)	9.8 ± 3.5	9.3 ± 3.3	9.8 ± 4.1	0.31	0.719
Neutrophil (%)	35.1 ± 16.4	32.7 ± 17.8	43.7 ± 20.0^a^ ^,^ ^b^	5.24	0.006
Lymphocyte (%)	53.1 ± 15.7	55.8 ± 17.4	44.6 ± 16.6^a^ ^,^ ^b^	6.30	0.002
Eosinophil (%)	2.1 ± 1.9	1.6 ± 1.3	1.5 ± 1.3	1.18	0.171
Hemoglobin (g/L)	116.9 ± 10.9	118.1 ± 27.7	108.4 ± 10.9^a^ ^,^ ^b^	4.16	0.017
Platelets (× 10^9^/L)	392.7 ± 128.2	398.8 ± 110.3	384.9 ± 127.2	0.17	0.844
Lactate dehydrogenase (U/L)	332.6 ± 71.5	359.7 ± 95.6	375.9 ± 168.5	1.73	0.181

*Note:* IQR, interquartile range.

^a^Compared to the mild group, *p* < 0.05.

^b^Compared to the moderate group, *p* < 0.05.

### 3.4. Relationship Between CC10 and CysLTs Protein Levels and Disease Severity in Infants Diagnosed With RSV‐Bronchiolitis

**FIGURE 2:**
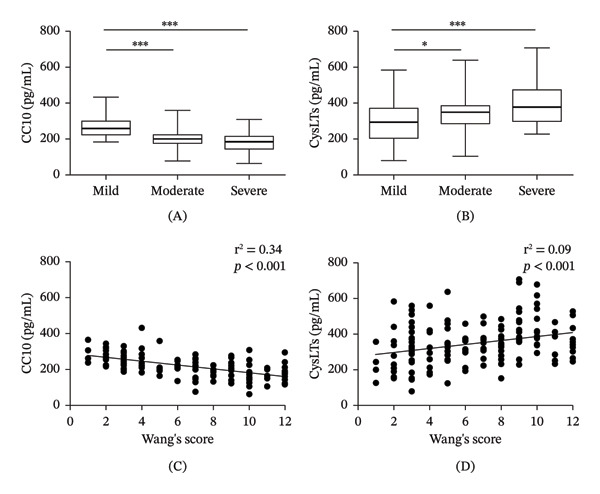
Relationship between CC10 and CysLTs protein levels and severity in infants with RSV‐bronchiolitis. The concentrations of CC10 and CysLTs in the NPAs were assessed using ELISA. (A) CC10 levels in children with different severities. ^∗∗∗^ *p* < 0.001, mild group (*n* = 61) compared to those of the moderate group (*n* = 56); ^∗∗∗^ *p* < 0.001, mild group (*n* = 61) compared to those of the severe group (*n* = 52). (B) CysLTs levels in children with different severities. ^∗^ *p* < 0.05, mild group (*n* = 61) compared with those of moderate group (*n* = 56); ^∗∗∗^ *p* < 0.001, mild group (*n* = 61) compared to those of the severe group (*n* = 52). (C) The correlation between CC10 and Wang’s scores in infants with RSV‐bronchiolitis. *r* ^2^ = 0.34, *p* < 0.001, *n* = 169. (D) The correlation between CysLTs and Wang’s scores in infants with RSV‐bronchiolitis. *r* ^2^ = 0.09, *p* < 0.001, *n* = 169.

The levels of CC10 were significantly lower in the severe group (184.1 ± 52.8 pg/mL) and in the moderate group (201.9 ± 45.5 pg/mL) compared to the levels in the mild group (266.5 ± 51.4  pg/mL; *p* < 0.001; Figure 2A). Pearson correlation analysis showed that CC10 protein level was negatively correlated with Wang’s score (*r* ^2^ = 0.34, *p* < 0.001; Figure 2C). Conversely, levels of CysLTs were significantly higher in the severe group (398.6 ± 123.7 pg/mL) and in the moderate group (342.9 ± 101.2 pg/mL) compared to the levels in the mild group (300.5 ± 122.7 pg/mL; severe group vs. mild group, *p* < 0.001; moderate group vs. mild group; *p* < 0.05; Figure 2B). CysLTs level was positively correlated with Wang’s score (*r* ^2^ = 0.09, *p* < 0.001; Figure 2D). It suggested that CC10 and CysLTs were related to severity in RSV‐bronchiolitis.

### 3.5. Clinical Characteristics of Children With Subsequent Wheezing After RSV Infection

Among 169 infants with RSV‐bronchiolitis, 27 cases were lost to follow‐up, and 142 cases completed follow‐up. Within a year after discharge, 47 cases (33.1%) had experienced subsequent wheezing, and 95 cases (66.9%) did not have subsequent wheezing. The age of children with subsequent wheezing (6.7 ± 4.7 months) was younger than that of children without subsequent wheezing (10.8 ± 8.1 months) (*t* = 2.67, *p* = 0.009). 63.8% (30 cases) of the children with subsequent wheezing had a history of allergies, which was significantly higher than 30.5% (29 cases) of the children without subsequent wheezing (*χ* ^2^ = 14.36, *p* < 0.001). The hospital stay of children with subsequent wheezing was 10.6 ± 5.2 days, which was longer than that of children without subsequent wheezing (8.4 ± 3.5 days) (*t* = 3.16, *p* = 0.002). The duration of ventilation was longer in children with subsequent wheezing (1.3 ± 0.2 days) than that of children without subsequent wheezing (0.4 ± 0.3 days) (*t* = 3.03, *p* = 0.003). There were no significant differences in sex, preterm delivery, feeding options, and laboratory examinations between children with and without subsequent wheezing (*p* > 0.05).

### 3.6. Relationship Between CC10 and CysLTs Protein Levels and Subsequent Wheezing in Infants Diagnosed With RSV‐Bronchiolitis

**FIGURE 3:**
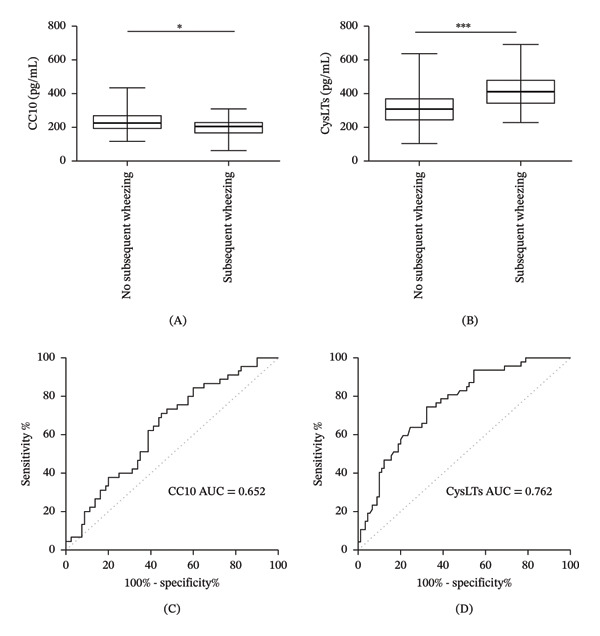
Relationship between CC10 and CysLTs protein levels and subsequent wheezing in infants diagnosed with RSV‐bronchiolitis. The concentrations of CC10 and CysLTs in the NPAs were assessed using ELISA. (A) CC10 levels in children with subsequent wheezing and without subsequent wheezing.^∗^ *p* < 0.05. (B) CysLTs levels in children with subsequent wheezing (*n* = 47) and without subsequent wheezing (*n* = 95). ^∗∗∗^ *p* < 0.001, children with subsequent wheezing (*n* = 47) compared to those without subsequent wheezing (*n* = 95). (C) The receiver operating characteristic (ROC) curve demonstrated the predictive role of CC10 for subsequent wheezing in children with RSV‐induced bronchiolitis. When CC10 levels were below 211.9 pg/mL, children were more likely to experience subsequent wheezing. At this level, the area under the curve was 0.652, with a sensitivity of 62.2% and a specificity of 61.3%. (D) The ROC curve demonstrated the predictive role of CysLTs for subsequent wheezing in children with RSV‐induced bronchiolitis. When CysLTs levels exceeded 335.3 pg/mL, children were more likely to experience subsequent wheezing. At this level, the area under the curve was 0.762, with a sensitivity of 78.7% and a specificity of 61.1%.

The protein levels of CC10 and CysLTs in NPAs were compared between children with and without subsequent wheezing, and the results are shown in Figure 3. The levels of CC10 were significantly lower in infants with subsequent wheezing than in those without subsequent wheezing (201.8 ± 48.6 pg/mL vs. 232.1 ± 52.1 pg/mL, *p* < 0.05, Figure 3A) following RSV infection. The levels of CysLTs were significantly greater in infants with subsequent wheezing than in those without subsequent wheezing (417.8 ± 141.6 pg/mL vs. 312.2 ± 124.6 pg/mL, *p* < 0.001, Figure 3B). Furthermore, CC10 and CysLTs performed well in predicting subsequent wheezing in RSV‐bronchiolitis. When CC10 levels were below 211.9 pg/mL, the area under the operating characteristic curve (AUC) reached 0.652, with a sensitivity of 62.2% and a specificity of 61.3% (Figure 3C). Conversely, when CysLTs levels exceeded 335.3 pg/mL, the AUC was 0.762, accompanied by a sensitivity of 78.7% and a specificity of 61.1% (Figure 3D). These findings indicate that the levels of CC10 and CysLTs proteins in NPAs have potential as predictive biomarkers for subsequent wheezing within 1 year after RSV‐induced bronchiolitis.

### 3.7. Predictors for Subsequent Wheezing in Infants With RSV‐Bronchiolitis Within 1 Year After Discharge

**FIGURE 4:**
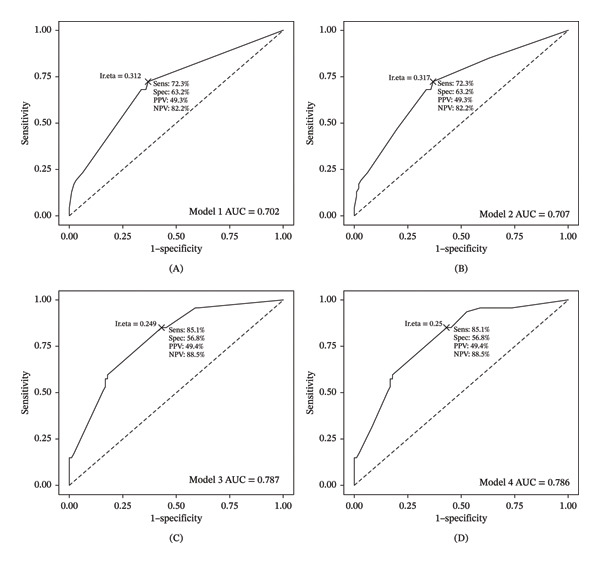
The receiver operating characteristic (ROC) curves for different models to predict subsequent wheezing after RSV infection. (A) ROC curves for Model 1 constructed with allergy history and duration of ventilation (AUC = 0.702, *p* < 0.001). (B) ROC curves for Model 2 constructed with allergy history and duration of ventilation + CC10 (AUC = 0.707, *p* < 0.001). (C) ROC curves for Model 3 constructed with allergy history and duration of ventilation + CysTLs (AUC = 0.787, *p* < 0.001). (D) ROC curves for Model 4 constructed with allergy history and duration of ventilation + CysTLs + CC10 (AUC = 0.786, *p* < 0.001).

The multivariate logistic regression model for predicting subsequent wheezing in RSV‐infected bronchiolitis is presented in Table 2. The results showed that CysLT levels, allergy history, and the duration of ventilation were predictors for subsequent wheezing within 1 year after RSV infection. Children with CysLTs protein levels greater than 335.3 pg/mL are more likely to experience subsequent wheezing (OR = 5.496). Children with a history of allergies are more likely to experience subsequent wheezing (OR = 3.466). The risk of subsequent wheezing increases with the duration of ventilation (OR = 1.364). Furthermore, we calculated the AUCs of four models to evaluate their performance. Model 1, constructed with allergy history and duration of ventilation, yielded an AUC of 0.702 with a sensitivity of 72.3% and a specificity of 63.2% (Figure 4A). Model 2, which additionally included CC10, achieved an AUC of 0.707 with a sensitivity of 72.3% and a specificity of 63.2% (Figure 4B). Model 3, incorporating CysLT instead, attained the highest AUC of 0.787 with a sensitivity of 85.1% and a specificity of 56.8% (Figure 4C). Model 4, containing both CC10 and CysLT, produced an AUC of 0.786 with a sensitivity of 85.1% and a specificity of 56.8% (Figure 4D). Consequently, Model 3 demonstrated the optimal performance. Thus, the model that combined allergy history, duration of ventilation, and CysLT achieved the highest AUC, suggesting that adding CysLT to clinical information improves the prediction of subsequent wheeze after RSV infection. The detailed statistics are presented in Table 3.

**TABLE 2:** The multivariate logistic regression model for predicting subsequent wheezing in RSV‐infected bronchiolitis.

Variables	*β*	SE	*Wald* *χ* ^2^	*p*	OR (95% CI)
Allergy history	1.243	0.421	8.708	0.003	3.466 (1.518∼7.916)
Duration of ventilation	0.310	0.137	5.097	0.024	1.364 (1.042∼1.786)
CYSTLs levels	1.704	0.465	13.449	0.000	5.496 (2.211∼13.633)

*Note:* CC10, club cell 10‐kDa protein; CysLTs, cysteinyl leukotrienes.

Abbreviations: CI, confidence interval; OR, odds ratio; SE, standard error.

**TABLE 3:** Predictive efficiency of different models for subsequent wheezing after RSV infection.

Model	Indicators	Sensitivity (95% CI)	Specificity (95% CI)	^+^LR	^−^LR	AUC (95% CI)	*p*
1	Allergy history and the duration of ventilation	72.3% (57.4%–84.4%)	63.2% (52.6%–72.8%)	1.96	0.44	0.702 (0.616–0.788)	< 0.001
2	Allergy history and the duration of ventilation + CC10	72.3% (57.4%–84.4%)	63.2% (52.6%–72.8%)	1.96	0.44	0.707 (0.617–0.798)	< 0.001
3	Allergy history and the duration of ventilation + CysLTs	85.1% (71.7%–93.8%)	56.8% (46.3%–67.0%)	1.97	0.26	0.787 (0.712–0.862)	< 0.001
4	Allergy history and the duration of ventilation + CC10 + CysLTs	85.1% (71.7%–93.8%)	56.8% (46.3%–67.0%)	1.97	0.26	0.786 (0.708–0.863)	< 0.001

*Note:* AUC, area under the ROC curve; CC10, club cell 10‐kDa protein; CysLTs, cysteinyl leukotrienes; ^+^LR, positive likelihood ratio; ^−^LR, negative likelihood ratio.

Abbreviation: CI, confidence interval.

## 4. Discussion

RSV infection is a major cause of wheezing in infants and young children, but currently there are no biomarkers that can predict the subsequent wheezing, nor are there established strategies to prevent and treat RSV‐induced recurrent wheezing. This study found that in children with RSV‐induced bronchiolitis, the level of CC10 protein was significantly reduced, and the level of CysLTs protein was significantly increased, closely related to the severity of the disease. Moreover, the lower the CC10 level is below 211.9 pg/mL, and the higher the CysLTs level is above 335.3 pg/mL, the greater the risk of subsequent wheezing within one year after discharge. In addition, allergic history and the duration of ventilation were also predictors for subsequent wheezing within a year after RSV infection.

CC10 is secreted by club cells, which possess self‐renewing capabilities in the tracheal and bronchial mucosa layer [12]. Circulating CC10 concentrations increased from childhood into adult life [13], and high levels of CC10 can inhibit excessive Th2‐type immune responses, helping to maintain airway homeostasis [14]. In mice, maternal smoking–induced CC10 reduction was accompanied by greater epithelial injury, whereas recombinant human CC10 restored the smoking‐impaired lung branching [15]. Children with allergic rhinitis have significantly reduced CC10 levels in nasal lavage fluid [16]. Low CC10 mRNA expression levels in bronchial epithelial cells were significantly associated with asthma susceptibility and asthma severity [17], and patients with chronic obstructive pulmonary disease and smokers have decreased CC10 in BALF, which is negatively correlated with the severity of the disease [18]. All the above suggest that CC10 has a protective role in airway injury diseases, and its level is related to the severity of the disease, consistent with the results of this study. Population‐based, multicohort studies have demonstrated that deficiencies in circulating CC10 are linked to asthma, characterized by frequent symptoms from childhood to mid‐adulthood, and also predict the persistence of asthma symptoms into adulthood [13]. This study found through follow‐up that when the level of CC10 protein is below 211.9 pg/mL, the likelihood of experiencing subsequent wheezing later increases. This suggests the predictive role of CC10 in wheezing associated with viral infections. However, multivariate analysis did not indicate its value in predicting subsequent wheezing.

CysLTs are downstream products generated by the action of lipoxygenase on AA, which is produced by the cPLA2 hydrolysis of cellular membrane phospholipids [8]. Previous studies found that after the administration of recombinant CC10 in RSV‐infected mice, the levels of CysLTs were reduced, accompanied by improvements in airway inflammation and AHR [9]. This confirms the downstream relationship between CC10 and CysLTs after RSV infection. Our study found that the level of CysLTs protein in the RSV infection group was significantly higher than that in the control group, and the level in severe cases was significantly higher than in mild cases, indicating that the level of CysLTs protein is positively correlated with the severity of the disease. CysLTs are elevated in asthmatic patients and bind to the receptor CysLT1 on target cells, leading to sustained bronchial constriction, mucus secretion, and edema, which are core mechanisms of asthma and important targets for leukotriene receptor antagonist (LTRA) treatment in asthma patients [8]. Our study found that individuals with CysLTs levels exceeding 335.3 pg/mL faced a risk of subsequent wheezing within a year after discharge, which was 5.496 times higher than that of others through multivariate analysis. This suggests that CysLT levels may serve as a potential biomarker for predicting subsequent wheezing following RSV infection.

It is well documented that RSV and many other respiratory viruses induce leukotriene synthesis [19], which is a major reason why children benefit more from LTRA therapy than adults. However, the efficacy of LTRA in treating virus‐triggered wheezing varies markedly among individuals [20], and indiscriminate widespread use is not only ineffective but may also increase the risk of serious neuropsychiatric side effects such as agitation, hallucinations, and depression [21]. Assessing the activation of the leukotriene pathway after RSV infection could provide a theoretical basis for giving LTRAs to children who experience subsequent wheezing but do not yet meet the criteria for asthma, thereby avoiding LTRA overuse. For example, children who show a marked drop in CC10 combined with a pronounced rise in CysLTs might be more prone to recurrent wheezing and eventual asthma and could therefore be the optimal candidates for early LTRA treatment.

Regarding the predictive role of epidemiological characteristics on subsequent wheezing, IgE‐related persistent wheezing can begin in infancy, with an increasing prevalence rate as age advances, and is associated with a personal and family history of atopy [22]. A history of wheezing episodes and maternal asthma were significantly associated with continued wheezing episodes [23, 24]. 90% of children with wheeze but no atopy lost their symptoms at school age and retained normal lung function at puberty. Food allergy in infancy is associated with a loss of lung function and asthma at age 6 years [25]. Blood eosinophil counts and percentages and allergic sensitization were significantly higher in children with recurrent severe wheeze than in children with a nonwheezing respiratory disease [26]. Early personal eczema is a significant risk factor for the development of asthma symptoms at 9–11 years old [27]. Children sensitized to multiple allergens are more likely to develop persistent atopic wheezing [28]. Respiratory support is an important part of treatment for infants with bronchiolitis, and in cases of severe illness where oxygen saturation cannot be maintained even with graded respiratory support, intubation may be necessary. Severe viral infections in early childhood that damage lung epithelial cells and the subsequent abnormal repair may be related to long‐term lung function impairment and recurrent wheezing [29].

In summary, the levels of CC10 and CysLTs in NPAs were related to severity in RSV‐bronchiolitis. The level of CysLTs, along with allergy history and the duration of ventilation, is expected to become a potential indicator for subsequent wheezing in children.

**Nomenclature**
AA: Arachidonic acid
AHR: Airway hyperresponsiveness
AUC: Area under the curve
BALF: Bronchoalveolar lavage fluid
CC10: Club cell 10‐kDa protein
CI: Confidence interval
cPLA2: Cytosolic phospholipase A2
CysLTs: Cysteinyl leukotrienes
ELISA: Enzyme‐linked immunosorbent assay
IQR: Interquartile range
LOX: Lipoxygenase
LTA4: Leukotriene A4
LTB4: Leukotriene B4
LTRA: Leukotriene receptor antagonist
NPAs: Nasopharyngeal aspirates
OR: Odds ratio
ROC: Operating characteristic curve
RSV: Respiratory syncytial virus

## Author Contributions

Study conception and design: Zhengzhen Tang and Sisi Chen.

Acquisition of data: Meng Deng and Qiujin Yuan.

Sample testing: Meng Deng and Qiujin Yuan.

Analysis and interpretation of data: Sisi Chen and Meng Deng.

Statistical analysis: Meng Deng and Qiujin Yuan.

Writing the manuscript: Sisi Chen and Meng Deng.

Review and accepting the manuscript: Zhengzhen Tang and Sisi Chen.

## Funding

The present study was supported by grants from the Science and Technology Research Program Project of the Chongqing Municipal Education Commission (Grant nos. KJQN202302818 and KJQN202402815), the Project of Chongqing Medical and Pharmaceutical College (Grant no. YGZZK2025106), and the Zunyi Science and Technology Cooperation (Project No. HZ‐2024–52).

## Ethics Statement

The acquisition and use of the NPAs were approved by the Institutional Review Boards of the Third Affiliated Hospital of Zunyi Medical University (the First People’s Hospital of Zunyi) (Permit number: 2022‐1‐45, Guizhou, China).

## Conflicts of Interest

The authors declare no conflicts of interest.

## Data Availability

The data that support the findings of this study are available on request from the corresponding authors. The data are not publicly available due to privacy or ethical restrictions.
